# Cardiopulmonary Parameters and Arterial Blood Gases During Etorphine-Medetomidine-Midazolam Immobilization in Free-Ranging Black Rhinoceroses (*Diceros bicornis*) Undergoing Electro-Ejaculation—A Preliminary Study

**DOI:** 10.3389/fvets.2021.740614

**Published:** 2021-12-02

**Authors:** Janine Meuffels, Imke Lueders, Henk Bertschinger, Ilse Luther-Binoir, Friederike Pohlin, Leandri Gerber, Brendan Tindall

**Affiliations:** ^1^Cryovault, Hemmersbach Rhino Force NCP, Tokai, South Africa; ^2^Department of Production Animal Sciences, Faculty of Veterinary Science, University of Pretoria, Pretoria, South Africa; ^3^GEOlifes, Animal Fertility and Reproductive Research, Hamburg, Germany; ^4^GEOSperm, Wildlife Reproduction and Biotechnology Services, Brits, South Africa; ^5^Research Institute of Wildlife Ecology, Department of Interdisciplinary Life Sciences, University of Veterinary Medicine Vienna, Vienna, Austria; ^6^Scientific Services Unit, Eastern Cape Parks and Tourism Agency, East London, South Africa; ^7^Robberg Veterinary Clinic, Plettenberg Bay, South Africa

**Keywords:** black rhinoceros, immobilization, heart rate, medetomidine, midazolam, arterial blood gases, blood pressure, semen collection

## Abstract

Conservation management interventions for the critically endangered black rhinoceros (*Diceros bicornis*) require immobilization, which offer opportunities for semen collection and cryopreservation to establish genetic reservoirs. In free-ranging rhinoceroses, a combination of the potent opioid etorphine and the tranquilizer azaperone is routinely used for chemical immobilization but is associated with muscle rigidity and severe cardiopulmonary changes. Additionally, azaperone inhibits semen emission. Seven free-ranging, male, sexually mature black rhinoceroses were immobilized with an alternative protocol consisting of 4.5 mg etorphine, 5 mg medetomidine, 50 mg midazolam and 2,500 IU hyaluronidase delivered remotely by darting from a helicopter. During the immobilization, electro-ejaculation was performed with a portable electro-ejaculator, and a species-specific rectal probe. Animals were observed for muscle tremors. Longitudinal changes in respiratory rate, heart rate and peripheral oxyhemoglobin saturation, measured at 5 min intervals, were assessed using a general mixed model. Non-invasive oscillometric blood pressure and arterial blood gas variables were measured at first handling and before reversal and compared using the Wilcoxon rank sum test. All animals were successfully immobilized, showed no muscle tremors, presented with normal heart rates and lactate concentration (<5 mmol/L), recovered uneventfully, but experienced acidemia, hypoxemia and hypercapnia. Induction time and total time in recumbency were 4.2 ± 0.41 and 38.4 ± 6.9 min, respectively. Electro-stimulation commenced after 11.7 ± 3.98 min and completed after 24.3 ± 6.65 min. Semen-rich fractions were successfully collected from six animals. Our observations indicate that etorphine-medetomidine-midazolam provides a promising immobilization protocol for free-ranging black rhinoceroses, that allows for successful electro-ejaculation.

## Introduction

In a recent assessment of the black rhinoceros population (1), the species was again classified as “critically endangered” by the International Union for Conservation of Nature (IUCN) with just over 3000 individuals in the wild—a result of the extensive poaching for horn (2).

As the numbers of individuals in the wild decrease, the preservation of gametes and their use in assisted reproductive technologies is gaining importance as additional conservation tools (3). Semen collection and cryopreservation allow to effectively preserve viable gametes in large numbers for future use in artificial insemination or ovum pick-up and embryo transfer by minimally invasive techniques and can be performed opportunistically during chemical immobilization for other management purposes (4). The development and improvement of species-specific protocols and equipment require sufficient sound knowledge of the reproductive anatomy and physiology, which for most wildlife species is based on access to animals in captivity (4). Electro-ejaculation (EE) as the most commonly used method for semen collection in wildlife species has been criticized to have animal welfare implications when used in conscious domestic animal patients. However, in wildlife species chemical immobilization is required for this procedure (4). Heart rates and serum cortisol levels were significantly lower in anesthetized domestic animals compared to conscious animals during and following EE (5).

Immobilization protocols for free-ranging black rhinoceroses are usually based on a combination of the potent opioid etorphine and a tranquilizer and/or sedative. Potent opioids result in rapid induction times and therefore a reduced risk of capture myopathy and injury (6, 7). However, they are associated with muscle rigidity, respiratory depression, hypertension, acidemia and hyperlactatemia (6, 8, 9). Azaperone is the most commonly added tranquilizer, but is reported to inhibit semen emission (10). An alternative protocol is therefore needed where semen collection is performed in free-ranging rhinoceroses. Etorphine in combination with medetomidine and midazolam was used repeatedly for intensive medical management in a black rhinoceros in captivity (11) and resulted in reliable immobilization and rapid recovery. The specific α_2_-adrenoceptor agonist medetomidine provides sedation, muscle relaxation and anxiolysis (12) and, additionally, was found to promote semen emission in other species (13, 14). Midazolam, a benzodiazepine receptor agonist, also provides sedation and muscle relaxation (15).

The aim of this study was to describe the changes in respiratory, cardiovascular and blood gas variables following immobilization of black rhinoceroses with an etorphine-medetomidine-midazolam combination for the purpose of EE.

## Materials & Methods

### Animals and Study Area

Seven free-ranging sexually mature (7–28 years) male black rhinoceroses (*Diceros bicornis*) were immobilized for ear-notching in a provincial nature reserve in the Eastern Cape, South Africa and were additionally subjected to electro-ejaculation.

### Chemical Immobilization

Rhinoceroses were located by direct observation from either a helicopter or fixed-wing aircraft. A combination of 4.5 mg etorphine (etorphine hydrochloride 9.8 mg/mL, Captivon; Wildlife Pharmaceuticals, Karino, South Africa), 5 mg medetomidine (20 mg/mL, Kyron Laboratories, Benrose, South Africa), 50 mg midazolam (midazolam hydrochloride 50 mg/mL, Dazonil; Wildlife Pharmaceuticals) and 2,500 IU hyaluronidase (lyophilised hyalase, Kyron Laboratories) was delivered by dart gun (Pneu-Dart 389, Williamsport, Pennsylvania, USA) from a helicopter into the gluteal muscles using 2.0 mL aluminum darts with 2.5-inch uncollared needles (Pneu-dart Type C, Pneu-dart, Inc., Williamsport). Immobilized rhinoceroses were approached quietly, blindfolded and earplugs applied to reduce external stimuli. The animals were kept in sternal recumbency throughout the immobilization. Time from darting to recumbency (induction time) and total time in recumbency (down time) were recorded.

To reverse the effects of etorphine, 50 mg naltrexone HCl (Trexonil 50 mg/ml, Wildlife Pharmaceuticals) were injected in an auricular vein. Medetomidine was reversed with 10–20 mg atipamezole HCl (5 mg/mL mg; Antisedan, Zoetis, Sandton, South Africa). One and two thirds of the dose were injected intravenously (auricular vein) and intramuscularly in the nuchal hump, respectively. The time from reversal until the animal was standing was recorded as recovery time.

### Monitoring, Blood Sampling, and Analyses

Blood samples (1 mL) were collected from the auricular artery into heparinized syringes (Arterial Blood Sampler Aspirator, Radiometer Medical ApS, Denmark) at first handling (t0) and before termination of immobilization (t_R_) and promptly analyzed using a portable blood gas analyzer (i-STAT Portable Clinical Analyzer) with cartridges (i-STAT CG4+ and Chem8+ cartridges, Zoetis, Germany). Blood pH, partial pressure of oxygen (PaO_2_), partial pressure of carbon dioxide (PaCO_2_) and lactate (Lac) were measured and base excess (BE), bicarbonate (${\text{HCO}}_{3}^{-}$) and hemoglobin oxygen saturation (SaO_2_) calculated by the analyzer at t0 and t_R_. At t0, sodium (Na), potassium (K), chloride (Cl), ionized calcium (iCa), glucose (Glu), urea (BUN), creatinine (Crea), and hematocrit (Hct) were additionally measured. All blood gas results were interpreted at a fixed temperature of 37°C.

Animals were observed for the presence of muscle tremors. At 5-min intervals from 5 min after recumbency (t_5_) until reversal of the immobilization, respiratory rate (f_R_) was determined by counting exhalation from the nostrils, and heart rate (HR) and peripheral oxygen hemoglobin saturation (SpO_2_) recorded with a pulse-oximeter (SunTech Vet30E, Morrisville, NC, USA) using a Y-lingual sensor (AccuVet) applied on a scarified edge of an ear. Attention was paid to not cause bleeding at the site of attachment. The same device (AccuVet), which is validated for indirect non-invasive measurement of blood pressure in horses, was used to measure mean (MAP), systolic (SAP) and diastolic (DAP) arterial blood pressures in the coccygeal artery as previously described (16) at t_0_ and t_R_. The 17 × 25 cm cuff was fitted around the tail, approximately 20 cm below the tail base and 20 cm above the right atrium and a single reading at each time point recorded.

### Electro-Ejaculation

EE was performed with a portable, battery-powered electro-ejaculator (El Toro 3, Electronic Research Group, South Africa) and a specifically designed rectal probe with three electrodes, which were positioned dorsal to the prostate as previously described (17). HR and f_R_ were recorded immediately before the start, at least 5 min after commencement and after completion of electro-stimulation. EE took a maximum of six sets of stimuli. Each set consisted of 8–10 stimulations lasting 3 s, with a 2-s pause in between. The voltage was slowly increased with each set of stimuli to reach a maximum of 10 V and 277 mA.

### Data Analyses

Statistical analysis was performed with the software R version 3.6.1 (18). Data were assessed for normality by calculating descriptive statistics and plotting of histograms. Median (range), were calculated for each parameter and time point (Tables 1, 2). A generalized linear model (fixed factor: time; random factor: individual rhinoceros) was used to assess the effect of time on HR, f_R_, and SpO_2_. Due to a small sample size, nonparametric analyses were used to compare arterial blood gas variables and non-invasive oscillometric blood pressure measurements at the beginning and end of immobilization, and HR and f_R_ before the onset, 5 min after commencement and after completion of EE, using the Wilcoxon rank sum test. Differences were considered significant when *P* ≤ 0.05.

**Table 1:** Heart rate (HR), respiratory rate (f_R_) and partial pressure of oxygen (SpO_2_) measured by pulse oximetry from the first measurement (t5) until maximum 50 mins (t50) and non-invasive oscillometric mean arterial (MAP), systolic (SAP) and diastolic (DAP) blood pressures at first handling (t0) and at reversal (t_R_).

**Time**	***n***	**Variable (Unit)**		
		**HR (beats/minute)**	**f_**R**_ (breaths/minute)**	**SpO_2_ (%)**
t5	*7*	60 (50–67)	4 (4–5)	80 (70–88)
t10	*7*	61 (50–67)	6 (4–7)	91 (79–97)
t15	*7*	56 (51–75)	6 (4–10)	92 (82–95)
t20	*7*	56 (52–80)	5 (4–7)	92 (83–96)
t25	*7*	57(52–77)	6 (4–8)	95 (90–97)
t30	*7*	58 (49–71)	5 (4–7)	90 (84–96)
t35	*4*	54 (32–66)	6 (4–6)	94 (79–95)
t40	*2*	59, 61	5	88, 94
t50	*1*	59	6	93
		**MAP (mmHg)**	**SAP (mmHg)**	**DAP (mmHg)**
t_0_		67 (32–178)	90 (54–226)	63 (28–169)
t_R_		161 (103–173)	199 (167–219)	147 (84–166)

*Data presented as median (min-max) and n = number of animals*.

**Table 2:** PH, partial pressure of carbon dioxide (PaCO_2_), pressure of oxygen (PaO_2_), base excess (BE), bicarbonate (${\text{HCO}}_{3}^{-}$), hemoglobin oxygen saturation (SaO_2_) and lactate (Lac) at first handling (t_0_) and reversal (t_R_) of seven black rhinoceroses anesthetized in this study with etorphine-medetomidine-midazolam; Blood chemistry parameters of seven black rhinoceroses at t_0_: sodium (Na), potassium (K), chloride (Cl), ionized calcium (iCa), Glucose (Glu), urea (BUN), creatinine (Crea), and hematocrit (Hct).

**Variable**	**Unit**	**Time**	
		**t_0_**	**t_R_**
pH		7.23 (7.14–7.38)	7.33 (7.28–7.36)
PaCO_2_	mmHg	56.8 (45–65)	49.6 (45–56)
PaO_2_	mmHg	63 (35–84)	66 (49–86)
${\text{HCO}}_{3}^{-}$	mmol/L	23.90 (15–29)	26.10 (22-28)
BE	mmol/L	−4 (−14–2)	0 (−5–2)
SaO_2_	%	87 (53–92)	92 (82–95)
Lac	mmol/L	1.5 (0.6–12.5)	0.53 (0.4–6.6)
Na	mmol/L	131 (125–133)	
K	mmol/L	4.8 (3.5–4.5)	
Cl	mmol/L	98 (96–103)	
BUN	mg/dL	7 (4–11)	
CREA	mg/dL	0.60 (0.06–0.80)	
Glu	mg/dL	85 (74–101)	
iCa	mmol/L	1.55 (1.46–1.60)	
Hct	%PCV	46 (40–50)	

*Data presented as median (range)*.

## Results

All animals were successfully immobilized, showed no muscle tremors, and recovered uneventfully. One animal needed a second dart with 2 mg etorphine, 5 mg medetomidine and 25 mg midazolam, presumably because of incomplete discharge of the first dart. His induction time was excluded from the induction time calculations. Mean induction time was 4.2 ± 0.4 min. Electro-stimulations started after 11.7 ± 4.0 (7–17) mins and were completed after 27.4 ± 8.9 (14–39) mins. The time from darting to reversal was 40.8 ± 7.5 (34–54) mins and total time in recumbency was 38.4 ± 6.9 (33-52) mins. Recovery after reversal was 1.7 ± 0.5 (1, 2) mins. Semen was successfully collected from six animals (86%) and first semen rich fractions were recovered as early as 40 s after the start of stimulation.

Descriptive analyses of variables measured in this study, are presented in Tables 1, 2.

Time had no effect on HR (*P* = 0.854) and f_R_ (*P* = 0.661). Median HR was 58 (50–67) before the start of stimulations, 61 (51–80) after at least 5 mins (6.7 ± 1.37 mins) and 60 (50–66) bpm when stimulation was ended. f_R_ was 5 (4–6), 6 (5–10) and 6 (4–7) breaths per minute during the same time points. HR and f_R_ immediately before stimulation did not differ from measurements after commencement (*P* = 0.173 and *P* = 0.157) and completion of stimulation (*P* = 0.395 and *P* = 0.335). Time had a significant effect on SpO_2_ (*P* = 0.008), which increased over time.

The pH, PaO_2_, SaO_2_ and BE were higher at t_R_ (*P* = 0.018, 0.018, 0.027 and 0.063 respectively) and PaCO_2_ and lac lower (*P* = 0.028 and 0.018) compared to t0. Lac was <5 mmol/L in all but one animal (Table 2). MAP, SAP and DAP did not differ significantly between t5 and t_R_ (MAP and DAP: *P* = 0.091; SAP: *P* = 0.063). SpO_2_ and SaO_2_ did not differ between t0 and t_R_ (*P* = 0.207; *P* = 0.916).

## Discussion

Immobilization with etorphine-medetomidine-midazolam resulted in short induction times similar to those previously reported in free-ranging black rhinoceroses immobilized with other etorphine-combinations (6, 8, 9) and uneventful recoveries. No muscle tremors were observed and lac was normal in all but one animals. However, rhinoceroses in this study initially presented with acidemia, hypoxemia and hypercapnia, which improved over the course of anesthesia. Indirect oscillometric blood pressure was hypertensive and the HR normal (normal heart rate range 32–42 bpm, reported from unrestrained standing white rhinoceroses) throughout the immobilization.

Capture of free-ranging rhinoceroses, especially when using helicopters, is linked with overexertion-related physiological changes including hyperthermia, hypoxemia, acidemia and hyperlactatemia. Potent opioids, although resulting in rapid induction, additionally are associated with severe side-effects like muscle rigidity, tachycardia, hypertension, respiratory depression and sequelae of the latter (6, 19). Hypoventilation resulting from μ-receptor-related effects of the etorphine including respiratory muscle rigidity, increased upper airway resistance and a central inhibitory effect on the respiration, as well as the pressure of the abdominal organs on the lungs and ventilation–perfusion mismatch, causes acidemia, hypoxemia and hypercapnia (9, 20–25). However, etorphine-induced pulmonary hypertension and its negative effects on gas exchange, was identified as main reason for the initial severe hypoxemia (26).

Hypoxemia and hypercapnia were comparable to etorphine-azaperone immobilized black rhinoceroses which were also positioned in sternal recumbency (6, 8). However, compared to rhinoceroses in those studies, our rhinoceroses had a lower f_R_ suggesting a higher tidal volume leading to a comparable alveolar minute ventilation and PaCO_2_. This is possibly due to improved muscle relaxation induced by medetomidine and midazolam decreasing chest wall rigidity and enhancing respiratory excursions resulting in a decrease in global ventilation-to-perfusion mismatch and improved gas exchange. The improved muscle relaxation compared to etorphine-azaperone and lack of muscle tremors may have decreased oxygen consumption and therefore CO_2_ production resulting in the comparable PaO_2_ and PaCO_2_ level (27). Further studies comparing the different anesthetic protocols under more standardized conditions are required to confirm these assumptions.

Acidemia, present in rhinoceroses immobilized with etorphine-combinations, is believed to be both of respiratory and metabolic (lactic acid) origin (6). The elevated PaCO_2_ concentrations at t0 indicate a respiratory acidemia in this study. No published lactate reference values for black rhinoceroses are available, however, five of the seven animals in this study had lactate concentrations that are within the normal range quoted for non-sedated white rhinoceroses (28). This suggests that lactate did not contribute to the acidemia in these six individuals. Only one animal showed a significantly elevated lactate concentration (12.49 mmol/L) together with the lowest recorded pH (7.136), but a normal PaCO_2_ (45 mmHg). Hyperlactatemia is associated with intense muscle activity following prolonged induction times and increased anaerobic metabolism with lactate production (29). The overall low lactate concentrations of the remaining animals including the one that was darted twice, suggest efficient induction with the drug combination used. Lateral recumbency, resulting in increased dead-space ventilation and more severe hypoxemia, has been associated with higher lactate values in previous studies (8). Sternal positioning employed in this study appeared to have a positive effect on ventilation.

Overall, blood gas values improved in all animals from t0 to t_R_, which suggests an improvement in ventilation during the immobilization. Similar results were obtained in other black rhinoceros studies that employed etorphine-based drug combinations (6). In a previous report on the repeated use of etorphine-medetomidine-midazolam in a black rhinoceros, immobilization and analgesia lasted for 34–78 mins without supplemental treatment (11). At t_R_, the effects of the anesthetic drugs were probably less pronounced than at t0, resulting in increased cardiac output, decreased pulmonary hypertension and therefore an improved gas exchange through enhanced pulmonary perfusion (26).

Systemic hypertension is a known side-effect of etorphine (6, 19) and will initially be exacerbated by the peripheral vasoconstriction caused medetomidine (12, 30). In this study, indirect blood pressures recorded at t5 varied greatly amongst the individuals and were only increased above the reference ranges for unsedated white rhinoceroses in three of the seven rhinoceroses (15). At t_R_, all rhinoceroses experienced hypertension, which could have been due to sympathetic activation caused by the EE. Unfortunately, continuous measurements during EE were not possible owing to the position of the probe handle close to the tail and thus the measuring cuff. Additionally, the method used has not been validated for rhinoceroses and, given the short, conical tail, the correct positioning of the cuff is rather challenging. Furthermore, the pronounced peripheral vascular resistance associated with the medetomidine at t_0_, may have resulted in incorrect readings and explain the large differences between individuals, no longer present t_R_.

Tachycardia is another common finding in rhinoceroses immobilized with etorphine-combinations (22), which was suggested to be a result of etorphine-induced upregulated sympathetic activity (31). Medetomidine on the other hand, is known to cause bradycardia as response to vasoconstriction as well as through a reduced sympathetic tone and suppression of the cardiovascular center (30, 32). This drug therefore may have compensated for the sympathomimetic action of etorphine and explain why heart rates remained within normal limits quoted for rhinoceroses in all individuals in this study (16).

EE had no effect on the HR and all animals remained immobilized indicating that the present immobilization protocol provided sufficient immobilization and analgesia. Continuous invasive blood pressure monitoring together with the measurement of serum catecholamine concentrations are required in future studies to truly investigate the sympathetic nociceptive response to EE in etorphine-immobilized black rhinoceroses. Finally, semen was collected successfully from six of seven individuals despite the limited time available, and with acceptable blood gas and acid base variables.

### Limitations

Data collection time points were pre-determined by the planned procedure as blood pressure measurements could not be repeated during the EE procedure. Additionally, the SunTech indirect blood pressure monitor has not been validated for the use in rhinoceroses. However, the equine settings were used and the paired measurements still provide information about the changes in blood pressure during the immobilization with etorphine-medetomidine-midazolam. The distance the animals moved from darting until recumbency was not estimated and may have influenced the results. Scarified SpO_2_ readings have not been validated in rhinoceroses. However, SpO_2_ readings were consistent with SaO_2_.

## Conclusions

The combination of etorphine-medetomidine-midazolam resulted in uncomplicated immobilizations, fast induction times and allowed for successful semen collection via electro-ejaculation. These preliminary results suggest that etorphine-medetomidine-midazolam is an acceptable immobilization protocol for use in free-ranging black rhinoceroses in general and for individuals undergoing EE.

## Data Availability Statement

The original contributions presented in the study are included in the article/supplementary material, further inquiries can be directed to the corresponding author/s.

## Ethics Statement

The animal study was reviewed and approved by University of Pretoria Animal Ethics and Research Committee (REC113-19).

## Author Contributions

JM, IL, and IL-B designed the experiment together with BT. BT took veterinary care of the rhinoceroses and collected the arterial blood samples. Data was collected and prepared by JM and analyzed by FP and JM. JM prepared the manuscript together with all co-authors. All authors approved the final manuscript.

## Funding

The study was funded by the non-profit company Rhino Force SA NPC. The funders had no role in study design, data collection and analysis, decision to publish, or preparation of the manuscript.

## Conflict of Interest

The authors declare that the research was conducted in the absence of any commercial or financial relationships that could be construed as a potential conflict of interest. The reviewer GZ declared a shared affiliation, though no other collaboration, with several of the authors JM and HB, to the handling Editor.

## Publisher's Note

All claims expressed in this article are solely those of the authors and do not necessarily represent those of their affiliated organizations, or those of the publisher, the editors and the reviewers. Any product that may be evaluated in this article, or claim that may be made by its manufacturer, is not guaranteed or endorsed by the publisher.

## References

[1] Emslie R. Diceros Bicornis. IUCN Red List Threat Species 2020 [Internet]. (2020).

[2] Emslie R. Ceratotherium Simum. IUCN Red List Threat Species 2020 [Internet]. (2020).

[3] Comizzoli P. Banking efforts and new advances in male fertility preservation for rare and endangered species. Asian J Androl. (2015) 17:640. 10.4103/1008-682X.153849 25966625 PMC4492057

[4] Prieto MT Hildebrandt TB Saragusty J. Sperm cryopreservation in wild animals. Eur J Wildl Res. (2014) 60:851–64. 10.1007/s10344-014-0858-4

[5] Orihuela A Aguirre V Hernandez C Flores-Perez I Vazquez R. Effect of anesthesia on welfare aspects of hair sheep (ovis aries) during electro-ejaculation. J Anim Vet Adv. (2009) 8:305–8.

[6] Morkel P Radcliffe RW Jago M Du Preez P Flaminio MJBF Nydam D V. Acid-base balance and ventilation during sternal and lateral recumbency in field immobilized black rhinoceros (diceros bicornis) receiving oxygen insufflation: a preliminary report. J Wildl Dis [Internet]. (2010) 46:236–45. 10.7589/0090-3558-46.1.236 20090037

[7] Kock M La Grange M Du Toit R. Chemical immobilization of free-ranging black rhinoceros (diceros bicornis) using combinations of etorphine (M99), fentanyl, and xylazine. J Zoo Wildl Med. (1990) 21:155–65.

[8] Radcliffe RW Morkel PV Jago M Taft AA Du Preez P Miller MA. Pulmonary dead space in free-ranging immobilized black rhinoceros (Diceros Bicornis) in Namibia. J Zoo Wildl Med. (2014) 45:263–71. 10.1638/1042-7260-45.2.263 25000686

[9] Fahlman A Edner A Wenger S Foggin C Nyman G. Pulmonary gas exchange and acid-base status during immobilisation of black rhinoceroses (Diceros Bicornis) in Zimbabwe. J S Afr Vet Assoc. (2016) 87:1–9. 10.4102/jsava.v87i1.1328 28155294 PMC6138189

[10] Meltzer DGA Van Vuuren M Bornman M. The suppression of electro-ejaculation in the chacma baboon (Papio Ursinus) by Azaperone. J S Afr Vet Assoc. (1988) 59:53. 3361563

[11] Monge Mora I Langan J Bailey R. Aitken-Palmer, C, Adkesson M, Tang K, Chinnadurai S. Repeated anesthesia in a black rhinoceros (diceros bicornis) to manage upper respiratory obstruction. J Zoo Wildl Med. (2018) 49:1041–6. 10.1638/2018-0095.1 30592926

[12] Sinclair M. A review of the physiological effects of alpha2-agonists related to the clinical use of medetomidine in small animal practice. Can Vet J. (2003) 44:885–97. 14664351 PMC385445

[13] Zambelli D Cunto M Prati F Merlo P. Effects of ketamine or medetomidine administration on quality of electroejaculated sperm and on sperm flow in the domestic cat. Theriogenology. (2007) 68:796–803. 10.1016/j.theriogenology.2007.06.008 17662381

[14] Kuczmarski HA Alves de. Barros M Souza de Lima LF Ferguson Motheo T Bento HJ Iglesias GI. Urethral catheterization after pharmacological induction for semen collection in dog. Theriogenology [Internet]. (2020) 153:34–8. 10.1016/j.theriogenology.2020.04.035 32417609

[15] Tallman JF Paul SM Skolnick P Gallager DW. Receptors for the age of anxiety: pharmacology of the benzodiazepines. Science. (1980) 207:274–81. 10.1126/science.6101294 6101294

[16] Citino SB Bush M. Reference cardiopulmonary physiologic parameters for standing, unrestrained white rhinoceroses (Ceratotherium Simum). J Zoo Wildl Med. (2007) 38:375–9. 10.1638/2006-0007R1.1 17939345

[17] Roth TL Stoops MA Atkinson MW Blumer ES Campbell MK Cameron KN. Semen Collection in rhinoceroses (rhinoceros unicornis, diceros bicornis, ceratotherium simum) by electroejaculation with a uniquely designed probe. J Zoo Wildl Med. (2005) 36:617–27. 10.1638/05-019.1 17312718

[18] R Development Core Team. R: A Language and Environment for Statistical Computing. Vienna, Austria: R foundation for statistical computing (2019).

[19] Mosing M Waldmann AD Sacks M Buss P Boesch JM Zeiler GE. What hinders pulmonary gas exchange and changes distribution of ventilation in immobilized white rhinoceroses (ceratotherium simum) in lateral recumbency? J Appl Physiol. (2020) 20:1140–9. 10.1152/japplphysiol.00359.2020 33054661

[20] Miller M Buss P Joubert J Mathebula N Kruger M Martin L. Use of butorphanol during immobilization of free-ranging white rhinoceros (Ceratotherium Simum). J Zoo Wildl Med [Internet]. (2013) 44:55–61. 10.1638/1042-7260-44.1.55 23505703

[21] Wenger S Boardman W Buss P Govender D Foggin C. The cardiopulmonary effects of etorphine, azaperone, detomidine, and butorphanol in field-anesthetized white rhinoceroses (Ceratotherium Simum). J Zoo Wildl Med. (2007) 38:380–7. 10.1638/2006-0038R.1 17939346

[22] Buss P Miller M Fuller A Haw A Wanty R Olea-Popelka F. Cardiovascular effects of etorphine, azaperone, and butorphanol combinations in chemically immobilized captive white rhinoceros (ceratotherium simum). J Zoo Wildl Med. (2016) 47:834–43. 10.1638/2015-0298.1 27691950

[23] Van Zijll Langhout M Caraguel CGB Raath JP Boardman WSJ. Evaluation of etorphine and midazolam anesthesia, and the effect of intravenous butorphanol on cardiopulmonary parameters in game-ranched white rhinoceroses (Ceratotherium Simum). J Zoo Wildl Med. (2016) 47:827–33. 10.1638/2015-0167.1 27691936

[24] Yaksh T Wallace M. “Opioids, Analgesia, and Pain Management,” in Goodman & Gilman's the Pharmacological Basics of Therapeutics, eds Brunton L, Chabner B, Knollman B. New York, NY: McGraw Hill Medical, 481–523 (2011).

[25] Pelosi P Croci M Ravagnan I Tredici S Pedoto A Lissoni A. The effects of body mass on lung volumes, respiratory mechanics, and gas exchange during general anesthesia. Anesth Analg. (1998) 87:654–60. 10.1213/00000539-199809000-00031 9728848

[26] Meyer RCR Hetem SS Mitchell D Fuller A. Hypoxia following etorphine administration in goats (capra hircus) results more from pulmonary hypertension than from hypoventilation. BMC Vet Res. (2015) 11:1–9. 10.1186/s12917-015-0337-5 25644810 PMC4322799

[27] Portas T. A review of drugs and techniques used for sedation and anaesthesia in captive rhinoceros species. Aust Vet J. (2004) 82:542–9. 10.1111/j.1751-0813.2004.tb11196.x 15478725

[28] Cole G Tordiffe A Steenkamp G. Assessment of a portable lactate meter for field use in the white rhinoceros (Ceratotherium Simum). Onderstepoort J Vet Res [Internet]. (2017) 84:1–10. 10.4102/ojvr.v84i1.1399 29227129 PMC8552300

[29] Buss P Olea-Popelka F Meyer L Hofmeyr J Mathebula N Kruger M. Evaluation of cardiorespiratory, blood gas, and lactate values during extended immobilization of white rhinoceros (Ceratotherium Simum). J Zoo Wildl Med. (2015) 46:224–33. 10.1638/2014-0089R.1 26056872

[30] England GCW Clarke KW. Alpha2 Adrenoceptor agonists in the horse—a review. Br Vet J. (1996) 152:641–57. 10.1016/S0007-1935(96)80118-7 8979422

[31] Boesch JM Gleed RD Buss P Hofmeyr M Tordiffe A Zeiler G. Effects of a supplemental etorphine dose on pulmonary artery pressure and cardiac output in immobilized, boma-habituated white rhinoceros (cerathoterium simum): a preliminary study. J Zoo Wildl Med. (2018) 49:849–55. 10.1638/2017-0120.1 30592907

[32] Knaus AE Muthig V Schickinger S Moura E Beetz N Gilsbach R. Alpha2-adrenoceptor subtypes – unexpected functions for receptors and ligands derived from gene-targeted mouse models. Neurochem Int. (2007) 51:277–81. 10.1016/j.neuint.2007.06.036 17664025

